# Computerized Cognitive Training with Older Adults: A Systematic Review

**DOI:** 10.1371/journal.pone.0040588

**Published:** 2012-07-11

**Authors:** Alexandra M. Kueider, Jeanine M. Parisi, Alden L. Gross, George W. Rebok

**Affiliations:** 1 Department of Mental Health, Johns Hopkins University Bloomberg School of Public Health, Baltimore, Maryland, United States of America; 2 Hebrew SeniorLife, Harvard Medical School, Boston, Massachusetts, United States of America; University of São Paulo, Brazil

## Abstract

A systematic review to examine the efficacy of computer-based cognitive interventions for cognitively healthy older adults was conducted. Studies were included if they met the following criteria: average sample age of at least 55 years at time of training; participants did not have Alzheimer’s disease or mild cognitive impairment; and the study measured cognitive outcomes as a result of training. Theoretical articles, review articles, and book chapters that did not include original data were excluded. We identified 151 studies published between 1984 and 2011, of which 38 met inclusion criteria and were further classified into three groups by the type of computerized program used: classic cognitive training tasks, neuropsychological software, and video games. Reported pre-post training effect sizes for intervention groups ranged from 0.06 to 6.32 for classic cognitive training interventions, 0.19 to 7.14 for neuropsychological software interventions, and 0.09 to 1.70 for video game interventions. Most studies reported older adults did not need to be technologically savvy in order to successfully complete or benefit from training. Overall, findings are comparable or better than those from reviews of more traditional, paper-and-pencil cognitive training approaches suggesting that computerized training is an effective, less labor intensive alternative.

## Introduction

Within 20 years, older adults will account for almost 25% of the U.S. population [1]. From a healthcare perspective, a major concern with an aging population is a higher prevalence of age-related impairment in cognitive function. This expanding aging population highlights the need to identify quick, effective, low-cost solutions to delay pathological cognitive decline associated with aging [2]. Developing interventions that can preserve cognitive function can also help to maintain quality of life and independence well into old age. With the help of new technology, novel cognitive training platforms, including computers and video games, can be readily disseminated to an older population.

Interest in training programs designed to improve cognitive abilities in older adults has been growing steadily in recent years. Ample evidence now suggests cognitive training interventions can improve cognitive performance in healthy older adults [3]–[6] and that these gains are robust up to five years after training [7]. A recent systematic review and meta-analysis reported that for healthy older adults, training improved performance in specific cognitive domains relative to control conditions [5].

Traditional cognitive training programs are delivered in individual or group format by a trained instructor, and differ primarily with regards to trained abilities (e.g., memory), length and frequency of training, and specific strategies practiced (e.g., method of loci for memory). Many traditional cognitive training programs require face-to-face contact (but see [8]), which entails identifying a convenient meeting location, coordinating schedules, and travel time. Further, traditional face-to-face training programs can be expensive. An hour of traditional cognitive training using a bachelor’s level trainer can cost $15 an hour, while an occupational therapist will charge up to $100 an hour [9]. These staff costs do not include the cost of equipment and materials. Given the importance of cognitive training for maintaining healthy cognitive function, cost-effective alternatives are needed. Computer-based cognitive interventions are a potentially cost-effective alternative to traditional training programs.

Additionally, not only can computer-based interventions be more cost effective, they can be more easily disseminated, reaching special populations that would otherwise not receive such interventions. Older adults who are home bound or live in an assisted living or nursing home facility and have limited access to transportation are difficult to recruit for traditional cognitive training programs. Computerized training programs could offer a more flexible, personalized approach to traditional cognitive training programs, allowing for easier access and dissemination to persons with access to technology. In addition, computerized programs provide real-time performance feedback and can adjust to the user’s ability level, keeping the activity engaging and fun. Poor adherence can be a challenge with traditional cognitive training programs (e.g., [10]). Computer and video games are designed to be fun and exciting and may provide motivation for older adults to stick with the training program.

In recent years the popularity of brain exercise products, currently a $300 million worldwide industry, has skyrocketed and is estimated to achieve between $2 and $8 billion in revenue by 2015 as the baby boomer generation continues to age [11]. The market is currently inundated with commercial brain exercise programs that claim to improve memory, attention, creativity, and delay Alzheimer’s disease and cognitive decline. However, few of these programs have been rigorously tested in empirical scientific studies with older adults, which is paramount to establish the efficacy of computerized training for aging individuals [10], [12].

Given the extensive body of research reporting older adults can benefit from cognitive training interventions and the personal computing revolution, the present systematic review summarizes the last 25 years of research on computerized training to address the following two questions: (1) What types of computerized training programs have been used to influence cognitive outcome measures among cognitively normal, community-dwelling older adults? (2) What is the strength of evidence that computerized cognitive training interventions influence cognition in this population? These are critically important questions in an expanding area of public health research because computerized training programs have the opportunity to capitalize on the increasing prevalence of personal computers among older adults and the increasing number of older adults to improve cognitive function and delay cognitive decline in later life.

## Methods

We conducted a review of studies on computerized training protocols for cognitively healthy older adults published or in press prior to July, 2011. To identify relevant studies, we searched computerized databases (PsycArticles, PsychInfo, Pubmed, SCOPUS) using combinations of the following key words for the population: *aging, aged, elderly, old, older adult*(*s*), *old,* and *oldest-old;* for cognitive function: *cognitive, cognitive abilities, cognition, memory, psychomotor speed,* and *speed of processing;* for interventions: *action games, computer(s), computerized training, enhancement, interactive gaming, intervention, video games, virtual reality,* and *training.* We also identified studies from reference lists in retrieved articles, unpublished dissertations, and conference abstracts. The supporting PRISMA checklist is available as supporting information (see Checklist S1).

To be included in the present review, the mean age of the study sample had to be, on average, at least 55 years of age at the time of training. Participants could not have a diagnosis of mild cognitive impairment or Alzheimer’s disease. Studies must have been published in English and have used a computerized approach targeted at any aspect of cognitive function. Computerized interventions included any study using an electronic game or task that involved participant interactions to produce visual feedback on a display device. Studies were excluded if the computerized training program sought only to evaluate the efficacy of the program itself (e.g., [13]), aimed to determine cognitive abilities that predicted success with training (e.g., [14]), did not include cognitive outcome measures (e.g., [15], [16]), results from younger adults could not be separated from older adults (e.g., [17]), or reviewed previous findings in the literature (e.g., [18]). In addition, only outcome measures of cognition were collected from eligible studies; outcome measures of everyday functioning, quality of life, and mood were excluded from this review. When outcomes were measured over several follow-up periods (e.g., post-training, 3 months, 6 months), only immediate post-training data were used to calculate effect sizes.

To synthesize study findings, studies were grouped into one of three categories based on the type of computerized training participants received: classic cognitive training tasks; neuropsychological software; or video games. Classic cognitive training tasks train specific aspects of cognition (e.g., processing speed or memory) using guided practice on standardized tasks. Neuropsychological software programs (e.g., NeuroPsychological Training, Colorado Neuropsychological Test) are designed to enhance multiple cognitive domains using a variety of tasks, can provide instant performance feedback, and are mostly self-guided, allowing participants to progress through tasks at their own pace. Video games can include electronic or computerized games in which the player manipulates images on a screen to achieve a goal. Within each of the three types of computerized training programs, studies were further grouped and reviewed according to interventions that targeted a single cognitive domain or interventions that targeted multiple cognitive domains.

### Effect Size Calculations

Effect sizes for a treatment effect reported by studies included partial eta-squared (η^2^), with values closer to 1.0 indicating a stronger effect size, and Cohen’s *d*. When not reported in a study, standardized Cohen’s *d* effect sizes were derived from the mean differences between scores on the post-training and pre-training cognitive outcome measures for the computer trained and control groups. Effect sizes (Cohen’s *d*) were standardized by dividing the mean differences for the computer trained and control groups by the pooled standard deviations of each cognitive outcome measure. For timed tests in which lower numbers indicate better performance, the direction of the association was reversed for ease of interpretation. Within the three categories of training median effect sizes were calculated for each cognitive domain.

## Results

Initially, 151 computerized training studies published between 1984 and 2011 were deemed relevant to the current review. Each study was reviewed and information pertaining to the study design, sample characteristics (e.g., age, cognitive status), cognitive outcomes, and the means and standard deviations of cognitive tests before and after training in the experimental and control groups were extracted.

**Figure 1:**
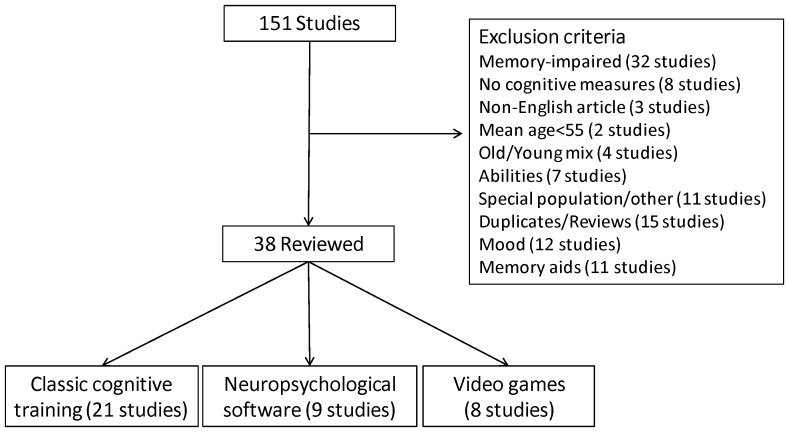
Identification of studies in the systematic review.

Based on the stated inclusion and exclusion criteria, 38 of the 151 publications were eligible for the current review (Figure 1). Common reasons for exclusion included pre-existing memory impairments in the sample (e.g., [19], [20]), average age less than 55 years (e.g., [15]), and not measuring cognitive function (e.g., [21]–[23]).

The 38 studies examined in this review used a total of 69 different cognitive measures encompassing global and domain-specific cognitive abilities. Tables 1, 2, and 3 summarize pertinent information from included studies in each of the three types of interventions. Examples of global cognitive measures included the Alzheimer’s Disease Assessment Scale-Cognitive Behavior section (ADAS-Cog), Repeatable Battery for the Assessment of Neuropsychological Status (RBANS), and the Wechsler Adult Intelligence Scale (WAIS). Domain-specific cognitive measures included memory (e.g., word list recall), executive functions (e.g., Trail Making Test Part B), information processing and psychomotor speed (e.g., Digit Symbol Substitution, Useful Field of View), and visual spatial functions.

**Table 1:** Classic Cognitive Training Studies Reported by Age Range, N, Intervention, Control, Duration, Significant Findings, and Effect Sizes.

Study	Age range	N	Intervention	Control	Duration	Significant Findings	Effect Sizes	
**Randomized Controlled Trials**								
Bherer, 2005	63–77	36	Dual task training: variable or fixed priority group	No contact	3 weeks: 2×/week for 60 min	Both IGs improved RT, no difference between groups.	Task set cost: IG: η^2^ = 0.60 CG: η^2^ = 0.01 Dual task cost: IG: η^2^ = 0.47 CG: η^2^ = 0.00	
Bherer, 2008	65–78	44	Dual task training: variable or fixed priority	No contact	3 weeks: 2×/week for 60 min	RT decreased and task accuracy improved in both IGs, no difference between groups.	Within modality RT: *d* range 3.84–6.32 Cross modality RT: *d* range 2.23–3.12	
Buschkuehl,2008	77–84	23	WM training	Exercise group	12 weeks: 2×/week for 45 min	IG improved on all WM and RT measures and several non-trained memory measures.	Block design: *d* = 1.34 Digit Span: *d* = 0.29 Visual memory: *d* = 0.26	
Dahlin, 2008	66–70	29	Executive function training	No contact	5 weeks: 3×/week for 45 min	IG improved training-specific executive function measure.	Digit symbol: *d* = 0.35 Digit span: *d* = 0.60 Letter fluency *d* = 0.37 N-back: *d* = 0.22	
Edwards, 2002	61–95	97	Processing speed training	No contact	2 weeks: 2×/week for 60 min	IG improved processing speed, CG improved verbal fluency/executive function.	RT: *d* = 0.13 UFOV: *d* = 1.09	
Edwards, 2005	70–82	126	Processing speed training	How to use computer/internet	5 weeks: 2×/week for 60 min	IG improved processing speed.	RT: *d* = 0.27 UFOV: *d* = 1.38	
Hinman, 2002	63–87	88	Biodex Balance System	No contact	4 weeks: 3×/week for 20 min	RT did not improve in the IG or CG.	*d* = 0.01	
Klusmann, 2010	70–93	259	Complex cognitive tasks	No contact	24 weeks: 3×/week for 90 min	IG improved memory and executive function.	Rivermead immediate *d* = 0.67; delay *d* = 0.57 FCSRT long: *d* = 0.52 EF: *d* = 0.45	
Mozolic, 2011	66–73	66	Selective visual and auditory attention training	Healthy aging lectures	8 weeks: 1×/week for 60 min	IG and CG improved executive function, WM, and RT, gains larger in IG.	RT: *d* = 0.52–0.57 Accuracy: *d* = 0.06–0.48 WM: IG: *d* = 0.48; CG: *d* = 0.46 Trail Making Test: *d* = 0.61 Symbol Digit: *d* = 0.34	
Roenker, 2003	62–79	95	Processing speed training	Driving simulator: driving skills; 2, 120 min sessions; or no contact	2 weeks for a total of 4.5 hours	IG UFOV performance equal to no contact group at post-test, driving group did not improve. IG improved RT.	RT: *d* = 0.68 UFOV: *d* = 2.19	
Slegars, 2009	65–73	201	Training: introduced and practiced with computers; Intervention: equipped with a computer and internet access, received no specific instructions	Participants in all no-intervention groups were instructed not to use a computer during the course of the study	Training: 2 weeks: 3, 4 hr sessions Intervention: 52 weeks	IG memory and EF compared to Training/No IG. Training/No IG was faster on executive function measure compared to the IG, No Training/No IG, and CG. Both training groups improved memory, both No Training Groups showed decreased performance.	Memory: *d* = 0.52 Task switching: *d* = 0.08 EF: *d* = 0.12-0.08	
**Non-Randomized and Pre-Post Designs**								
Vance, 2007	70–81	159	Processing speed training, discussed how speed of processing was related to everyday activities	How to access and use the Internet/email	3–68 weeks (M = 12 weeks): ten 60 min sessions	IG larger gains on processing speed and attention. IG and CG improved visuo-spatial abilities, psychomotor speed, and memory.	UFOV: *d* = 1.62	
Bisson, 2007	69–79	24	Virtual reality: exercise program or biofeedback: dynamic balance training	None	10 weeks: 2×/week for 30 min	Both IGs improved RT.	Biofeedback *d* = 0.69 Virtual reality *d* = 0.22	
Cassavaugh, 2009	65–79	21	Attention, visuo-spatial WM, and manual control tasks	None	8, 90 min sessions	Improved EF, attention, processing speed, and increased accuracy.	EF: *d* = 4.12 Visuospatial ability: *d* = 2.71 Selective attention *d* = 4.09	
Finkel, 1989	65–78	280	Computer Assisted Instruction: using method of loci mnemonic; amount of training not specified	Mnemonics skills sessions and classroom mnemonics training	Total of 14hours for 2 hrs/day	IG and CG improved memory, no difference between groups.	IG: *d* = 0.45 CG: *d* = 0.39	
Jennings, 2005	69–72	46	Repetition lag memory training: recollection or recognition practice	No contact	3weeks: 2×/week for 60 min	Recollection IG improved memory, psycho-motor speed, and EF accuracy.	Processing speed: Recollect: *d* = 1.30; Recognition: *d* = 0.58 WM: Recollect: *d* = 2.30; Recognition: *d* = 0.41 N-back: Recollect: *d* range 1.19–3.92; Recognition: *d* range 0.89–2.82	
Lajoie, 2003	70.85	24	Balance training	None	8 weeks: 2×/week for 60 min	IG improved RT.	*d* = 1.17	
Li, 2008	70–77	41	Spatial WM training	No contact	12 weeks: 45 daily sessions for 15 min	IG improved spatial WM and executive function.	Spatial WM: *d* = 0.88 RT: *d* = 0.96	
Lustig, 2008	67–82	32	Integrative processing training (modified version of repetition lag training): Integrated Sentences or Strategy Choice group	None	3 weeks: 8, 110 min sessions	Both IGs improved training-specific memory measure, only Integrated Sentences group improved on a non-trained memory measure. Both IGs improved executive function, gains larger in Integrated Sentences group.	Executive function: Integrate: *d* = 0.36; Strategy: *d* = 0.27	
Ralls, 1997	52–87	60	Logical reasoning and spatial ability training:, basic computer course	Two courses on Myers Briggs work styles; basic computer course	Training: 3, 120 min sessions. Computer course: 6 weeks: 1×/week for 90 min	IG improved spatial orientation.		
Wadley, 2006	65–94	84	Lab or home-based Processing speed training	How to use computer/internet or No Contact	5 weeks: 2×/week for 60 min	Both IGs improved processing speed, no difference between groups.	RT: *d* = 0.51 UFOV: *d* = 1.50	

Abbreviations: CG: Control Group; FCSRT: Free and Cued Selective Reminding Test; IG: Intervention Group; RT: Reaction Time; UFOV: Useful Field of View; WM: Working Memory.

**Table 2:** Neuropsychological Software Studies Reported by Age Range, N, Intervention, Control, Duration, Significant Findings, and Effect Sizes.

Study	Age range	N	Intervention	Control	Duration	Significant Findings	Effect Sizes
**Randomized Controlled Trials**							
Blackford, 1989	51–78	45	Einstein Memory Trainer: focused on names and faces, method of loci, peg word, important dates and phone numbers; or Classroom Instruction: Einstein Memory manual	Problem-solving and conceptual skills cognitive rehabilitation software, computer games; or no contact	8 weeks: 2×/week	Classroom IG improved more than computer IG on visuo-spatial abilities, computer CG improved more than no contact controls. Classroom IG improved more than no contact controls on delayed measure of visuo-spatial ability.	Visuospatial ability: Classroom: *d* = 1.24; computer: *d* = 0.19; computer control: *d* = 0.59 Delayed visuo-spatial ability: classroom: *d* = 1.11
Bottiroli, 2009	60–73	44	NeuroPsychological Training	Wait-list	3 weeks: 1×/week for 120 min	IG improved training-specific memory measures. IG and CG improved on transfer memory measures, larger gains in IG.	Recognition: *d* = 0.56 Face-name learning: *d* = 0.43 Memory: *d* = 0.68 Place-word learning: IG: *d* = 1.01; CG: *d* = 0.36
Eckroth-Bucher, 2009	70–87	37	Sound Smart and Captain’s Log programs, paper and pencil based activities	No contact	6 weeks: 2×/week for 45 min	Non-impaired IG improved logical memory.	*d* = 0.62
Mahncke, 2006	60–87	187	Memory, sensation, motor control, and cognition tasks	Educational lectures; or no contact	8–10 weeks: 5×/week for 60 min	IG improved on task-specific measures, gains generalized to non-trained measures of memory.	Processing speed: *d* = 7.14 RBANS: *d* = 0.25 Word recognition: *d* = 2.92 WM: *d* = 3.00
Peretz, 2011	60–77	155	CogniFit Personal Coach®	Computer games (Tetris, Memory Simon and pairs, puzzles, snake, target practice, math triangle)	12 weeks: 3×/week for 20–30 min	IG and CG improved focused and sustained attention, memory recognition, and mental flexibility; only IG improved memory recall, visuo-spatial learning/WM, and executive function. Participants with lower baseline scores benefited the most.	Focused attention: IG: *d* = 0.63; CG: *d* = 0.29 Sustained attention: IG: *d* = 0.35; CG: *d* = 0.37 Memory recognition: IG: *d* = 0.50; CG: *d* = 0.33 Memory recall: IG: *d* = 0.48 Visuospatial learning: IG: *d* = 0.51 Visuospatial WM: IG: *d* = 0.43 Executive function: IG: *d* = 0.42 Mental flexibility: IG: *d* = 0.39; CG: *d* = 0.27
Rasmusson, 1999	69–86	46	Colorado Neuropsychological Test memory tasks	Audiotape: *Mega Memory, Neuro-psychology of Memory Power*; or memory course; or Wait-list	9 weeks: 1×/week for 90 min	Computer and memory groups improved memory. Performance decreased on prospective memory in IGs compared to CG.	Episodic memory: *d* = 0.94 Verbal memory: *d* = 0.31
Rebok, 1996	71–82	12	Colorado Neuropsychological Test memory tasks	None	9 weeks: 1×/week for 90 min	IG improved on implicit and explicit memory.	Implicit memory: ?Z = 2.08 Explicit memory: ?Z = 0.83
Smith, 2009	69–82	487	Posit Science Brain Fitness Program	Educational DVDs	8–10 weeks: 4–5×/week for 60 min	IG improved auditory memory/attention, memory, and processing speed.	Processing speed: *d* = 0.87 RBANS: *d* = 0.23 Word recall: *d* = 0.27 Delayed word recall: *d* = 0.20 Digit span: *d* = 0.26 Letter number sequencing: *d* = 0.23
**Pre-post Design**							
Berry, 2010	62–81	30	Lab or home-based Posit Science Sweep Seeker visual training	No contact	3–5 weeks: 3–5×/week for 40 min	IG improved on trained and untrained perceptual tasks.	Perception: medium: *d* = 0.85; high: *d* = 0.88 Discrimination: *d* = 0.45

Abbreviations: CG: Control Group; IG: Intervention Group; WM: Working Memory.

**Table 3:** Video Game Studies Reported by Design, Age Range, N, Intervention, Control, Duration, Significant Findings, and Effect Sizes.

Study	Age range	N	Intervention	Control	Duration	Significant Findings	Effect Sizes
**Randomized Controlled Trial**							
Goldstein, 1997	72–85	22	SuperTetris	No contact	5 weeks: at least 300min/week; playing time varied: 25.5–36.5 hrs	IG improved RT. IG and CG improved executive function, no difference between groups.	*d* = 1.11
**Non-Randomized and Pre-post Designs**							
Ackerman, 2010	50–71	78	Wii *Big Brain Academy*	None	4 weeks: 5×/week for 60 min	IG improved on task-specific fluid, crystallized and perceptual speed measures.	*d* = 1.70
Basak, 2008	63–75	39	*Rise of Nations*	No contact	4–5weeks: 3×/week for 90 min	IG improved memory, executive function, and visuo-spatial abilities.	Executive control: η^2^ = 0.42 N-back: η^2^ = 0.10 Memory: η^2^ = 0.09 Reasoning: η^2^ = 0.11
Belchoir, 2008	67–84	58	UFOV or *Medal of Honor*	Tetris or no contact	2 weeks: 2–3×/week for 90 min	UFOV IG improved processing speed more than no contact controls, no difference between *Medal of Honor* and Tetris groups.	UFOV: *d* = 1.62; Tetris: *d* = 0.36
Clark, 1987	57–83	14	Pac Man or Donkey Kong	No contact	7 weeks: 120 min/week	IG improved RT	*d* = 0.33; 0.56
Drew, 1986	61–78	13	Atari Crystal Castles	Contact with researcher	8 weeks: 2×/week for 60 min	IG improved psychomotor speed and global cognition.	WAIS: *d* = 0.77 WAIS verbal: *d* = 0.39 WAIS performance: *d* = 0.71
Dustman, 1992	62–71	60	Breakout, Galazian, Frogger, Kaboom, Ms. Pacman, Pengo, and Qix	Movie viewing or no contact	11 weeks: 3×/week for 60 min	IG improved RT. IG and CGs improved executive function.	RT: *d* = 0.97 Attention: *d* = 0.25
Torres, 2008	70–86	43	QBeez, Super Granny 3, ZooKeeper, Penguin Push, Bricks, Pingyn, memory games	Muscle relaxation or no contact	8 weeks: 1×/week	IG showed less cognitive decline compared to CG.	*d* = 0.67

Abbreviations: CG: Control Group; IG: Intervention Group; RT: Reaction Time; UFOV: Useful Field of View.

### Classic Cognitive Training Tasks

Twenty-one studies used cognitive domain-specific programs including speed of processing, memory, attention, and perception in older adults 61 to 95 years of age (Table 1). Across all classic cognitive training studies the median effect size for each cognitive domain was 0.69 for reaction time, 1.30 for processing speed, 0.89 for working memory, 0.39 for executive function, 0.52 for memory, 0.39 for visual spatial abilities, and 0.57 for attention.

#### Duration

Training sessions lasted from two weeks to 24 weeks, ranging from daily to three times weekly sessions. Two studies only required participants meet a specific time requirement for training which ranged from 10 hours [24] to 12 hours [25]. Another study allowed participants to use their personal computers as long as they liked over the course of a year [26].

#### Reaction time (3 studies)

Reaction time is the amount of time needed to process and respond to a stimulus and is critical for handling information [27], [28]. Studies that implemented computerized balance training programs, in which participants received real-time visual postural feedback, reported conflicting results about the benefits of training for reaction time. Bisson et al. [29] and Lajoie [30] reported improved simple reaction times after training (biofeedback group: *d* = 0.69, virtual reality group: *d* = 0.22 [29]; and *d* = 1.17 [30]), whereas Hinman found no improvement in simple reaction time in the intervention group compared to controls.

#### Processing speed (5 studies)

Processing speed is the ability to quickly process information. Results of five studies [9], [24], [32]–[34], suggested speed of processing interventions, which varied in duration from two to 12 weeks, significantly improved processing speed scores (Useful Field of View) with effect sizes (Cohen’s *d)* ranging from 1.09 [32] to 2.19 [34]. Three studies [9], [32], [33] reported improvements on visual spatial abilities (Road Sign Test) with effect sizes ranging from 0.13 [32] to 0.51 [9], but no impact of training on executive function (Trail Making Test Part B) or psychomotor speed (Digit Symbol Substitution Test). Roenker et al., [34] reported improvements on choice reaction time (*d* = 0.68 [34]), while Vance et al. [24] reported larger improvements in the intervention group on a measure of visual sensory function and attention (*d* = 0.23) compared to controls.

#### Memory (5 studies)

Memory is the ability to retain, store, and recall information [35]. There are many different types of memory (e.g., recall, recognition, episodic, verbal, visual, and working memory) and various training strategies that can be used to enhance it (e.g., rehearsal, categorization, visualization, pegword, method of loci). Finkel and Yesavage [36] compared computer-assisted instruction with traditional classroom-based mnemonic training. Participants in both groups improved on memory (mean word list recall: intervention: *d* = 0.45; control: *d* = 0.39), and the differences between the two groups were not significant (p = .26). Findings suggest computer-assisted instruction, which is less labor intensive, may serve as a viable alternative to more traditional classroom-based training.

Two studies [37], [38] examined the effects of repetition lag training over a period of three weeks. Repetition lag training focuses on learning a list of words and involves a variety of retrieval and encoding processes responsible for memory recall and recognition. Although both studies reported multiple measures of verbal memory (California Verbal Learning Test [37]; shopping list memory, face-name association [38]) were not impacted by training, results in other cognitive domains varied.

Conflicting results were observed on processing speed and working memory measures. Jennings and colleagues [37] found a positive impact of training on processing speed (Digit Symbol Substitution Task: Recollection group: *d* = 1.30; Recognition group: *d* = 0.58), as well as working memory (Self-Ordered Pointing Task: *d* = 2.30; N-back task: effect sizes ranged from 1.19 to 3.92 for the Recollection group and 0.89 to 2.82 for the Recognition group), whereas Lustig and Flegal [38] reported no benefits in either processing speed (pattern comparison test) or working memory (Self-Ordered Pointing Task), but positive results on executive function (Trail Making Test Part B; Integrated Sentence group: *d* = 0.34, Strategy Choice group: *d* = 0.27).

Two studies, both with 12 week duration [39], [40], examined the effects of working memory training on older adult’s cognition, and reported positive results on working memory tasks. Buschkuehl and colleagues [39] reported improved performance on two training-specific measures of working memory and reaction time. Additionally, improved performance transferred to non-trained working memory tasks (block span: *d* = 1.34; digit span: *d* = 0.29) and visual memory (visual free recall: *d* = 0.26). However, there was no benefit of training on a verbal memory measure (verbal free recall).

Similarly, Li et al. [40] reported positive results for participants who received spatial working memory training. Training significantly improved performance on practiced spatial working memory tasks (task accuracy: *d* = 0.88) and reaction time (*d* = 0.96) and resulted in positive transfer to working memory measures (spatial three-back, numerical-two back, and numerical three-back, all versions of the N-back task).

#### Executive function (3 studies)

Executive function encompasses a broad spectrum of abilities including planning, cognitive flexibility, and abstract thinking skills. Results of three studies which trained executive function over a span of three [41], [42] to five weeks [43] varied, and included improvements in reaction time [41], [42] and measures of executive function [43]. Dahlin et al. [43] reported participants in the intervention group showed improved performance after training on the recall of numbers, letters, colors, and spatial locations, and tasks requiring the continuous updating, categorization, and association of presented material, suggesting executive function abilities are modifiable in older adults following computerized training. On non-trained cognitive tasks, modest improvements were observed on measures of working memory (digit symbol: *d* = 0.35; digit span backwards: *d* = 0.60) and phonemic fluency (letter fluency: *d* = 0.37), and may suggest that executive function training has limited generalizability to other cognitive domains [43].

In two separate studies [41], [42] participation in dual task training to improve executive control skills, which provided continuous, individualized feedback, improved accuracy on the dual tasks, and decreased reaction times in the variable and fixed priority intervention groups with effect sizes ranging from 2.23 (dual-cross modality transfer task) to 6.32 (dual-within modality transfer task) [42]. There was no significant difference in improvement between the two intervention groups (η^2^ = 0.01), suggesting that both were equally effective. Results from the two studies suggest that dual-task training reduced reaction time, task-set (η^2^ = 0.60) [41], and dual-task costs (η^2^ = 0.47) [41] relative to controls.

#### Attention (1 study)

Selective attention is the process by which an individual directs or focuses on specific auditory or visual stimuli in the environment. Modality-specific selective attention training (i.e., visual and auditory), in which participants were taught strategies to reduce the impact of sensory modalities on task performance, was used for eight weeks [44]. Training resulted in larger improvements on attention tasks (divided attention effect sizes ranged from 1.20 to 4.07; selective attention effect sizes ranged from 0.20 to 1.64), as indicated by decreased reaction time interference (effect sizes ranged from 0.52 to 0.57) and increased accuracy (effect sizes ranged from 0.06 to 0.48).

Training-specific improvements transferred to other cognitive domains as well. Working memory significantly improved after training in both the intervention (1-back portion of the N-back; *d* = 0.48; 2-back: *d* = 0.25) and control groups (*d* = 0.46). The effect of training on executive function was mixed: the intervention group improved more than the control group on a measure of executive function/processing speed (Symbol Digit Modalities Test: *d* = 0.34; Trail Making Test: *d* = 0.61), but there was no impact of training on executive function (Stroop Color Word Test) or verbal memory (Hopkins Verbal Learning Test).

#### Multiple cognitive domains trained (4 studies)

Based on ample evidence suggesting little transfer of cognitive training effects to untrained cognitive abilities [10], some investigators have trained multiple cognitive domains with a single intervention to better characterize transfer effects and generalizability of findings across domains. Four studies [25], [26], [45], [46] used interventions that targeted multiple cognitive domains including aspects of memory, executive function, visual spatial ability, and processing speed. Twelve hours of training with tasks that depended on attention and visual spatial ability significantly executive function (N-back: *d* = 4.12) and visual spatial abilities (tracking tasks: *d* = 2.71; selective attention task accuracy: *d* = 4.09) [25].

A 24-week computer course training complex cognitive tasks targeting multiple cognitive abilities improved performance on some measures of memory (Rivermead Behavioral Memory test immediate recall: *d* = 0.67 and delayed recall: *d* = 0.57; Free and Cued Selective Reminding Test (FCSRT) long delay recall: *d* = 0.52), and executive function (Trail Making Test part B: *d* = 0.27) [45]. No significant differences were observed for measures of immediate memory (FCSRT short delay), executive function (Stroop Color Word Test), or verbal fluency.

Ralls [46] administered a training intervention which focused on improving logical reasoning and spatial ability prior to a six-week computer course on the basics of computer use. Participants who received the intervention significantly improved on a measure of spatial orientation (paper folding test), compared to controls. However, results indicated no effect of training on measures of logical reasoning after controlling for pre-training cognitive ability.

One study assessed the impact of computer and internet use on older adult’s cognition [26]. Training consisted of a basic computer course in which participants were taught how to operate a computer and perform simple tasks (e.g., word processing), while the intervention equipped participants with a computer and Internet access for one year with no specific usage instructions. Results indicated participants who received both the Training and the Intervention (the intervention group) showed higher total scores on a measure of memory (Visual Verbal Learning Test: *d* = 0.52) and better flexibility scores on a measure of task switching (Concept Shifting Task: *d* = 0.08) over time compared to participants in the Training, No Intervention group. Participants in the Training, No Intervention group were faster on a measure of executive function (Stroop Color Word Test) compared to all the other groups with small effect sizes ranging from 0.12 (No Contact Control) to 0.08 (No Treatment/No Intervention group). No combination of intervention or training impacted processing speed (Letter-Digit Substitution Test) or reaction time (Motor Choice Reaction Test).

### Neuropsychological Software

The second body of research included nine studies that used neuropsychological software designed to test and enhance multiple domains of cognition in older adults aged 60 to 94 years (Table 2). Across all neuropsychological software studies the median effect size for each cognitive domain was 4.00 for processing speed, 0.45 for working memory, 0.39 for executive function, 0.56 for memory, 0.59 for visual spatial abilities, and 0.36 for attention.

#### Duration

Training sessions lasted from three weeks to 12 weeks with sessions ranging from once weekly to five times weekly.

#### Memory (3 studies)

Two studies that used explicit and implicit memory tasks from the Colorado Neuropsychology Test (CNT) software for nine weeks to assess the effect of training on memory reported positive outcomes. Rasmusson and colleagues [47] reported participants who received training improved on a measure of episodic memory (Rivermead Behavioral Memory Test; *d* = 0.94) and verbal learning and memory (Hopkins Verbal Learning Test; *d* = 0.31). Rebok et al. [48] also used memory tests from the CNT software and reported positive results after training. Standardized scores on the CNT were used to show improvement across training. Improvements were larger for implicit memory tests (ΔZ = 2.08) compared to explicit memory tests (ΔZ = 0.83).

Blackford [49] used the self-paced Einstein Memory Trainer software to examine the effect of memory training via computerized instruction versus a traditional group format. The Einstein Memory Trainer program focused on name and faces, method of loci, peg words, important dates, and phone numbers, was self-paced, and gave performance feedback. The computerized intervention group used the Einstein Memory Trainer software, whereas the traditional classroom intervention group learned from a software manual. The computer control group used cognitive rehabilitation software designed to improve problem-solving and conceptual skills. On a measure of visual spatial ability (Wechsler Memory Scale Visual Reproduction test (WMS-VR)), the classroom intervention (*d* = 1.24), computer intervention (*d* = 0.19), and computer control group (*d* = 0.59) improved more than no-contact controls. On a delayed measure of visual spatial ability (delayed WMS-VR), the classroom intervention improved more than the no-contact controls (*d* = 1.11). Training did not affect executive function (Trail Making Test part A and B) or measures of memory (California Verbal Learning Test; Name-Face Association Test). There was no evidence to support the superiority of computer training to group-based training.

#### Multiple cognitive domains trained (6 studies)

Sweep Seeker, a stand-alone module in Posit Science InSight software packages, was used for five weeks to train visual perception and working memory in a lab or home-based setting [50]. Results suggested both interventions were equally effective at training visual perception and working memory (*p* = 0.36 for group differences). Training resulted in improved performance on trained perceptual tasks of medium (*d* = 0.85) and high difficulty (*d* = 0.88). On untrained tasks, perceptual discrimination improved significantly (*d* = 0.45) for trained participants, suggesting benefits of training transferred to untrained perceptual tasks.

Two studies implemented training protocols using a program designed by Posit Science for eight to ten weeks which focused on improving multiple cognitive abilities [4], [6]. In both studies, training improved measures of processing speed (*d* = 7.14 (4]; *d* = 0.87 [6]) and auditory memory and attention (RBANS Auditory Memory/Attention: *d* = 0.25 [4]; *d* = 0.23 [6]). Additionally, training improved several other areas including verbal memory (Rey Auditory Verbal Learning Test total word recall: *d* = 0.27 and delayed word recall: *d* = 0.20 [6]; forward word recognition span: *d* = 2.92 [4]) and working memory (digit span: *d* = 3.00 [4]; digit span backwards: *d* = 0.26; letter number sequencing: *d* = 0.23 [6]). However, training did not appear to affect episodic memory (Rivermead Behavioral Memory Test) [6].

The Integrated Cognitive Stimulation and Training Program (ICSTP) was designed to incorporate both paper-and-pencil based training activities with two computer software programs, Sound Smart and Captain’s Log, to simultaneously train multiple cognitive abilities [51]. For non-impaired intervention group participants, performance improved on a measure of logical memory (Wechsler Memory Scale-Logical Memory total recall: *d* = 0.62) after training. No previous studies have integrated traditional paper-and-pencil based methods and computer technology in training.

The NeuroPsychological Training (NPT) program was designed to stimulate cognitive domains related to attention, language, memory, perception, and reasoning [52]. Post-training performance on practiced tasks (recognition figures list: *d* = 0.56 and face-name learning task: *d* = 0.43) was higher in the intervention group compared to the control group. Training effects were noted on a measure of memory (paired-associate recall test: *d* = 0.68) for participants in the training group, but not for wait-list controls. Both groups improved on a transfer task, which measured place-word learning, but gains were larger in the intervention group (*d* = 1.01) compared to controls (*d* = 0.36).

One study compared the effect of 12 weeks of training, using either the CogniFit Personal Coach, a personalized cognitive training software, or classic computer games (e.g., Tetris, snake, puzzles, Memory Simon, memory pairs) that significantly engaged cognitive processing [53]. Participants in the CogniFit Personal Coach group improved on all eight cognitive domains measured (focused attention: *d* = 0.63; sustained attention: *d* = 0.35; memory recognition: *d* = 0.50; memory recall: *d* = 0.48; visual spatial learning: *d* = 0.51; visual spatial working memory: *d* = 0.43; executive function: *d* = 0.42; mental flexibility: *d* = 0.39), while participants in the classic computer games group showed improvement in only four domains (focused attention: *d* = 0.29; sustained attention: *d* = 0.37; memory recognition: *d* = 0.33; mental flexibility: *d* = 0.27). Participants with lower baseline cognitive function benefited most from CogniFit training.

### Video Games

Eight studies investigated the effects of video games as a means of improving the cognitive abilities of older adults aged 50 to 87 years (Table 3). Across all video game studies the median effect size for each cognitive domain was 0.77 for reaction time, 0.72 for processing speed, 0.25 for executive function, 0.21 for attention, and 0.69 for global cognition.

Unlike neuropsychological software, most video games (with the exception of Nintendo Wii’s Big Brain Academy) were not originally designed to improve various aspects of cognition and thus are less targeted towards a specific cognitive domain. Commercially available video games included Big Brain Academy, Rise of Nations, and Medal of Honor, while classic video/computer games included Pac Man, Donkey-Kong, Tetris, and Atari video games (e.g., Breakout, Crystal Castles, Galazian, Frogger, Kaboom). One study used a combination of classic cognitive training tasks and video games [54].

#### Duration

Training sessions lasted from two weeks to 11 weeks, with sessions ranging from twice weekly to five times weekly. Two studies only required participants to meet a specific time requirement for training which ranged from 2 hours [55] to 5 hours per week [56]. One study had no time requirements and allowed participants to play video games as long as they liked [57].

#### Processing speed (1 study)

Clark and colleagues [55] studied the effect of playing Pac-Man or Donkey-Kong on processing speed for seven weeks. Results indicated at post-test, the mean reaction time for the intervention group was faster compared with controls on both compatible (responded to stimuli directly in front of their finger; *d* = 0.33) and incompatible (responded to stimuli opposite of their finger; *d* = 0.56) tasks and did not result from a speed-accuracy trade off.

#### Attention (1 study)

To assess the impact of video games on visual attention, older adults were assigned to one of four conditions: UFOV (Useful Field of View) training, Medal of Honor video game, Tetris (a video game control), or a no-contact control group [54]. Medal of Honor, a first person shooter game, has been shown to improve a number of visual and attentional abilities in younger adults and was the main intervention under study, whereas Tetris was selected because previous studies reported little or no effect on visual attention performance of college students [58]. After training the UFOV group improved significantly more on a processing speed measure compared to the Medal of Honor (UFOV task: *d* = 0.73), Tetris (*d* = 0.72) and no-contact control (*d* = 0.98) groups. While the Medal of Honor group significantly improved on a processing speed measure compared to the Tetris (*d* = 0.72) and no-contact control (*d* = 0.31) groups.

#### Multiple cognitive domains trained (6 studies)

Six studies that trained older adults to play various video games (e.g., SuperTetris, Rise of Nations, Crystal Castles, Big Brain Academy) over a span of three to 11 weeks reported positive results in multiple cognitive domains (e.g., reaction time, multiple types of memory, executive function), but results varied significantly between studies.

Two independent studies [56], [59] reported improved reaction time (*d* = 0.97 [59]; *d* = 1.11 [56])after using Nintendo SuperTetris for five weeks [56] or a variety of Atari games (Breakout, Galaxian, Frogger, Kaboom, Ms. Pacman, Pengo, and Qix) for 11 weeks [59]. Conflicting results were reported for a measure of executive function (Stroop Color Word Test). Nintendo Super Tetris [56] appeared to improve executive function abilities in both intervention and control groups (*d* = 0.37), while a variety of Atari games had no effect [59]. In addition, Dustman and colleagues [59] reported participants in the intervention and control groups improved on an executive function/processing speed measure (Symbol Digit Modalities Test: *d* = 0.25). The intervention did not affect psychomotor speed, verbal or visual memory (Benton Visual Retention Test), or visual motor tracking (Trail Making Test Part B).

In contrast to the previous studies [59] that found no impact of video game training on a measure of executive function (Stroop Color Word Test), a more recent study reported positive results in executive control for participants who played Microsoft Game Studios Rise of Nations, a real-time strategy game thought to improve executive functioning, for four to five weeks when compared to no-contact controls [60]. After training, older adults significantly improved on tasks related to executive control (η^2^ = 0.42), working memory (N-back: η^2^ = 0.10), visual short-term memory (Visual Short Term Memory task: η^2^ = 0.09), and reasoning abilities (Raven’s Advanced Matrices: η^2^ = 0.11). Video game training also had a positive effect on task-switching (η^2^ = 0.17), with performance peaking after 23.5 hours of training. No effect of training was seen on measures of visual spatial abilities (Functional Field of View Task, attentional blink task, operation span task) or reaction time.

Not only do video games improve specific cognitive domains for older adults, evidence suggests they can affect global cognitive functioning as well. Two studies that used a variety of video games for eight weeks reported improved global cognitive functioning [57], [61]. Atari’s Crystal Castles, an arcade video game, was hypothesized to improve perceptual motor skills and cognitive functioning of older adults [61]. After training, participants significantly improved on global measures of cognition (WAIS-R full scale IQ: *d* = 0.77, verbal: *d* = 0.39, and performance: *d* = 0.71 subtests) and psychomotor speed (*d* = 0.88; Rotary Pursuit: *d* = 0.61), whereas controls showed no improvements [61]. In a study by Torres [57], global cognitive performance improved for participants who played a variety of video games (QBeez, Super Granny 3, ZooKeeper, Penguin Push, Bricks, Pigyn). After training, participants showed less cognitive decline, as indicated by lower scores on a measure of global cognition (ADAS-Cog: *d* = 0.67), than both active and no-contact control groups.

One study used a Nintendo Wii video game specifically marketed for brain training, Big Brain Academy [62]. After four weeks, participants significantly improved on Wii tasks as illustrated by a large effect size (*d* = 1.70). Although participants showed significant improvement on Wii specific tasks, these positive effects did not transfer to measures of crystallized, fluid, or perceptual speed ability tests.

## Discussion

This systematic review summarized the types of computerized training that have been studied in older adults, and explored evidence of training benefits for computerized training among older adults. Based on this review, all three approaches to computerized training – classic cognitive training tasks, neuropsychological software, and video games – appear to hold promise for improving cognitive abilities in cognitively normal, community-dwelling older adults who have a higher risk of cognitive decline as they age. Studies that used classic cognitive training and neuropsychological software had the most rigorous designs, with 57% (n = 12) of classic cognitive training and 89% (n = 8) of neuropsychological software studies using a randomized controlled trial. In addition, studies using these two approaches had larger samples sizes relative to the video game studies (Table 4).

**Table 4:** Descriptive Statistics of Computerized Cognitive Training Studies.

	Classic cognitive tasks	Neuropsychological software	Video games
	(n = 21)	(n = 9)	(n = 8)
Design, n. (%)			
Randomized controlled trial	12 (57.1)	8 (88.9)	1 (12.5)
Non-Randomized controlled trial	3 (14.3)	0 (0.0)	1 (12.5)
Pre-post	6 (28.6)	1 (11.1)	6 (75.0)
Sample Size			
Mean (SD)	87.4 (77.1)	115.9 (151.6)	40.9 (23.6)
Median (range)	53 (259)	45 (475)	41 (65)

Abbreviation: SD: Standard Deviation.

Effect sizes reported in this systematic review are comparable to or better than those reported in non-computerized cognitive training interventions. A meta-analysis of classic memory training interventions reported an average standardized pre-post training gain of 0.73 standard deviations [63]. A more recent meta-analysis, which analyzed the effect of memory training on specific memory abilities, reported effects sizes ranging from 0.06 (face-name delayed recall outcome measures) to 1.10 (short-term memory outcome measures) when comparing healthy older adults in treatment conditions to controls [5]. Given the similarity between computer-based and traditional cognitive training interventions, our findings justify pursuing computer-based interventions in the future.

### Classic Cognitive Training Tasks

Based on the evidence reviewed, classic cognitive training interventions improved reaction time, processing speed, working memory, executive function, memory, visual spatial ability, and attention. For reaction time effect sizes ranged from 0.22 [29] to 1.17 [30] with a median effect size of 0.69; for processing speed effect sizes ranged from 0.54 [9] to 3.28 [34] with a median effect size of 1.30; for working memory effect sizes ranged from 0.25 [44] to 3.92 [37] with a median effect size of 0.89; for executive function effect sizes ranged from 0.08 [40] to 6.32 [42] with a median effect size of 0.39; for memory effect sizes ranged from 0.26 [39] to 0.67 [45] with a median effect size of 0.52; for visual spatial ability effect sizes ranged from 0.13 [32] to 4.09 [25] with a median effect size of 0.39; for attention effect sizes ranged from 0.20 to 4.07 [44] with a median effect size of 0.57. Together, these findings suggest the benefits of such computerized training programs are highly comparable to more traditional approaches.

While significance tests were not performed, working memory, executive function, and processing speed appear to be more amenable to change with classic cognitive training tasks. These domains had the largest effect sizes when compared with those of reaction time, memory, visual spatial abilities, and attention.

### Neuropsychological Software

Although results varied according to the specific intervention, overall, neuropsychological software programs appear to positively impact cognitive performance. With the exception of Blackford [49] all reviewed studies found benefits of training on memory. Effect sizes ranged from 0.20 [6] to 2.92 [4] with a median effect size of 0.56. Visual spatial abilities improved across two studies with effect sizes ranging from 0.19 to 1.24 [49] with a median effect size of 0.59. Across four studies, measures of working memory improved after training with effect sizes ranging from 0.23 [6] to 3.00 [4] with a median effect size of 0.45. Processing speed effect sizes ranged from 0.87 [6] to 7.14 [4] with a median effect size of 4.0.

Overall, neuropsychological software appears to be least effective in the domains of attention and executive function. While the domains of memory and visual spatial ability are more amenable to change with neuropsychological software.

### Video Games

Based on the evidence reviewed, video games appear to be an effective means of enhancing reaction time, processing speed, executive function, and global cognition in older adults. Effect sizes for reaction time ranged from 0.33 [55] to 1.11 [56] with a median effect size of 0.77; for processing speed effect sizes ranged from 0.31 to 0.98 [54] with a median effect size of 0.72; for executive function effect sizes ranged from 0.11 to 0.42 [60] with a median effect size of 0.25; and for global cognition effect sizes ranged from 0.39 to 0.71 [61] with a median effect size of 0.69.

Video game training appeared to have the largest impact on measures of reaction time and processing speed as these cognitive domains had the largest effect sizes. Results were less consistent across studies on measures of executive function and memory and may be explained by the differences in the cognitive tests used to measure these abilities. It is also possible that video game interventions are not an effective means of changing executive function and memory in older adults.

Computer-based cognitive training programs offer several advantages over traditional cognitive training programs, including the ability to individualize training according to the individual’s needs and to reach home-bound or institutionalized older adults. Additionally, computerized programs could be a more cost-effective alternative that offers the possibility of more widespread dissemination among older adults. Because computerized interventions require less face-to-face training, administration costs could be significantly reduced. Computerized cognitive interventions also offer a self-paced individualized experience, allowing individuals to focus only on areas that need improvement. This individualized format also could benefit older adults who experience performance anxiety in a more traditional group-format intervention.

The results from individual studies suggest older adults do not need to be technologically savvy to benefit from training. Many of the older adult participants in the reviewed studies had no prior experience with the technologies (i.e., video games, computers) used in the intervention studies and yet they were still able to benefit from these novel approaches. Previous research has shown participants’ prior use of computers was not significantly associated with acquisition of computer skills during training sessions, suggesting older adults can benefit from novel technologies [64].

Despite common misperceptions older adults do not enjoy learning to use new technology, perceptions of the computerized training programs were positive for the older adults who completed computerized training [65], [66]. In spite of many older adults reporting anxiety about using unfamiliar technology at the beginning of training, most reported high levels of satisfaction after training was completed. Some older adults stated they could use their new video game skills to connect more with their grandchildren [57]; whereas others were very willing to learn to use video games and believed they could be a positive form of mental exercise [54].

It is important to note that inconsistencies may be due to several factors not related to the actual training program itself, including different cognitive outcome measures and modifications of the training program. However, several limitations of this review need to be mentioned. First, the large variability in the types of training techniques used as well as length of protocols makes it difficult to determine the optimal type and dose of computer-based interventions that are the most effective. Second, due to the wide variety of cognitive measures used, control variables in multivariable models, and training interventions, we were unable to conduct a traditional meta-analysis. Meta-analysis assumes effect estimates all have the same underlying meaning, which is violated in the present set of studies because of the wide variability in the type and length of training, as well as cognitive outcome measures used to report results. Thus, estimated effects from each study are not equivalent and should not be combined using meta-analysis.

While it is possible that publication bias may lead to inflated effect sizes, every effort was made to locate and include results from unpublished studies. Three dissertations were included in the current review [46], [49], [54] as well as unpublished results presented at a conference [57]. Finally, studies which included older adults with mild cognitive impairment (MCI) were excluded from the current review. As the current diagnostic criteria of MCI only became well known until after 1999 [67], articles published prior to 2000 may have inadvertently included MCI participants. However, even though MCI criteria were not defined until 1999 [67], this group of individuals was well known and described in the literature as those with incipient dementia and isolated memory impairment among other things [68]–[70].

Older adults are the now fastest growing segment of Internet users [71]. According to a 2010 Pew Internet and American Life survey [72], 78% of adults aged 50–64 years and 42% of adults older than 65 years of age use the Internet. This is a sharp increase from 2000 when only 50% of adults 50–64 years and 15% of adults older than 65 years of age used the Internet [72]. As ownership of personal computers continues to grow and more older adults have access to the Internet [73], cognitive training programs need to take fuller advantage of these outlets to improve cognitive function and delay cognitive decline in later life.

While there is evidence that computerized cognitive interventions are beneficial in cognitively health community-dwelling older adults, there is need for future research. More well-designed randomized controlled trials with larger samples sizes are necessary to confirm these results. Computerized training may be a lonely individual activity and long-term adherence to such programs may be quite limited. Future studies should investigate this aspect of computer training. Furthermore, future studies should examine the efficacy and feasibility of web-based programs geared towards older adults.

## Supporting Information

Checklist S1 **PRISMA Checklist.**
(DOC)
